# Incidental findings on imaging exams: what is the essential nature of radiology?

**DOI:** 10.1590/0100-3984.2019.52.2e3

**Published:** 2019

**Authors:** Fernando Ide Yamauchi, Hilton Muniz Leão Filho, Manoel de Souza Rocha, W. W. Mayo-Smith

**Affiliations:** 1 Department of Radiology and Oncology, University of São Paulo School of Medicine, São Paulo, SP, Brazil. Email: fernando.yamauchi@einstein.br.; 2 Vice Chair of Radiology, Brigham & Women's Hospital, Harvard Medical School, Boston, MA, USA.

Radiology has played an essential role in the diagnosis and treatment of many medical conditions in the last three decades. The higher spatial resolution of modern computed tomography (CT) and magnetic resonance (MR) scanners has resulted in early detection of diseases, as well as providing an opportunity to understand and possibly change the natural history of several neoplasms.

The benefits of early detection have to be balanced against the large number of incidental findings^(^^1^^)^. In many instances, such findings are not true neoplasms and are not associated with any clinical morbidity but still generate high costs for the health care system, due to long-term follow-up and expensive diagnostic tests, as well as causing anxiety for patients. For example, tiny pancreatic cysts are a very common finding on routine MR scans of the abdomen, especially when thin-slice MR-cholangiopancreatography sequences are performed. The follow-up suggested by clinical and surgical consensus is strict and seems overestimated^(^^2^^)^. However, which of those cysts will develop into a malignant neoplasm and how long that process takes are still unanswered questions and create opportunities for future research. Incidentally detected adrenal thickening and small adrenal masses are other examples of findings that may trigger extensive clinical and laboratory work-ups, as well as requiring follow-up periods of up to five years^(^^3^^)^.

The burden of health care costs is concerning worldwide, and there are opportunities to improve patient care while reducing medical costs. When analyzing incidental findings, it is important to keep in mind our primary aim as radiologists, which is not chasing incidental findings but rather diagnosing disease or facilitating the diagnostic process^(^^4^^)^. We may have missed the essential nature of radiology and started "chasing ghosts"^(^^5^^)^. Our real purpose (*telos* in Aristotle's philosophy) should always be to safeguard patient welfare, and care should be taken when reporting incidental findings that could potentially divert us from delivering the best patient care. Nevertheless, we do realize that some incidental findings are truly important and are sometimes even more significant than the main clinical issue.

There are several opportunities for radiology to take the lead in this discussion. First, we should increase the added value of a radiology report, focusing on the clinical questions to be answered. Understanding the background of each patient may enable us to weight our reports regarding incidental findings. How important is a renal cyst in a terminal oncologic patient? Second, radiology committees may help to standardize imaging workflow in several scenarios, and radiologists could act more as consultants than as reporters^(^^6^^)^. We need radiology to be more efficient and pragmatic, focusing on patient management rather than "radiology by the book". Radiology reports should be focused less on the differential diagnosis and more on suggestions for the referring physician. It may be more relevant to suggest a biopsy or even surveillance on a small renal mass than to guess the correct histology of the lesion. Third, quantitative imaging may also reveal some important value "hidden" in CT examinations ("opportunistic data"), such as the assessment of sarcopenia and abdominal fat^(^^7^^-^^9^^)^, as well as the burden of abdominal aorta calcification and cardiovascular risk^(^^10^^)^. We can go even further, looking at bone density and the risk of vertebral fracture^(^^11^^)^, together with liver fat content, liver iron content, and even hepatic fibrosis^(^^12^^-^^14^^)^. Finally, the use of artificial intelligence and big data may allow us to gather all relevant data even before the radiology report is issued, helping us deliver a more robust and meaningful report^(^^15^^)^. If we do so, we may have rediscovered our *telos*.
